# Dental Erosion in Patients with Gastroesophageal Reflux Disease (GERD) in a Sample of Patients Referred to the Motahari Clinic, Shiraz, Iran

**Published:** 2014-03

**Authors:** G. Alavi, AA. Alavi, M. Saberfiroozi, AH. Sarbazi, M. Motamedi, Sh. Hamedani

**Affiliations:** a Post Graduate Student, Department of Oral and Maxillofacial Pathology, School of Dentistry, Shiraz University of Medical Sciences, Shiraz, IRAN; b Biomaterial Research Center, Dept. of Operative Dentistry, School of Dentistry, Shiraz University of Medical Sciences, Shiraz, Iran; c Gastroenterohepatology Research Center, Nemazee Hospital, School of Medicine, Shiraz University of Medical Sciences, Shiraz, IRAN; d Dept. of Prosthodontics, School of Dentistry, Aja University of Medical Sciences, Tehran, IRAN; e Dept. of Operative Dentistry, School of Dentistry, Shiraz University of Medical Sciences, Shiraz, IRAN; f Dental Research Development Center (DRDC), School of Dentistry, Shiraz University of Medical Sciences, Shiraz, IRAN

**Keywords:** Dental erosion, Gastroesophageal reflux disease, Risk factor

## Abstract

**Statement of Problem: **Systematic reviews of the literature show that the dental erosion is associated with the gastroesophageal reflux disease (GERD).The prevalence of the problem may not be exclusively similar in different countries.

**Purpose:** The purpose of this study was to investigate the association of gastro-esophageal reflux disease (GERD) with dental erosion in a sample of Iranian population regarding the standing difference in the Iranian oral hygiene and diet.

**Materials and Method:** 140 patients with the average age of 30 to 50 years old comprised the study group. The participants were already eligible for the endoscopic examination, diagnosed by their gastroenterologist. All patients completed a detailed questionnaire regarding the medical and dental situations. After completing the questionnaire and before endoscopy, dental examination was performed by two blinded dentists.The endoscopy was then performed by a gastroenterologist and the patients were divided into three groups of healthy, suspected to GERD, and positive GERD. Data were collected and analyzed by Chi- Square test. The cross tabulation test was performed to compare the qualitative variants and discover the correlations. The statistical significance was adopted as: p < 0.05.

**Results:** The prevalence of dental erosion in GERD patients (22.6%) was found to be higher than the suspected (5.3%) and the healthy (7%) individuals.

**Conclusion:** This study declared the GERD patients are at higher risk of developing dental erosion compared to the healthy individuals in a sample of Iranian population.

## Introduction

Gastroesophageal reflux disease (GERD) depicts a condition that involves all the individuals who are prone to the physical complications of gastroesophageal reflux or otherwise, their health and life quality is significantly impaired by problems associated with the symptoms of this disease [1].

Retrograde movement of the acid from the stomach into the esophagus is normally controlled by lower esophageal sphincter through an anti-reflux mechanism. The failure of this coordination may lead to the chronic condition recognized as gastroesophageal reflux disease (GERD) [1-2]. GERD manifestations in the oral cavity are reported as dental erosions, malodour, burning sensation, mucosal ulceration, loss of taste, xerostomia and sometimes increased salivary flow [3].

The incidence of GERD considerably increases after 40 years of age. An extensive geographical disparity in the prevalence is also reported [1, 4].

Roughly, 20-40% of the adult population in Western countries suffer from the known symptoms of GERD [4-5]. GERD has some dental complications and the most common is the increased dental erosion [6-8].

Dental erosion is an irreversible loss of tooth enamel, dentin and cementum due to the chemical reactions not induced by bacterial actions [9]. Dental erosion is caused by a combination of extrinsic and intrinsic etiologic factors. The extrinsic factors include acidic foods, acidic beverages, sports drinks and chewable vitamin C tablets [1]. Intrinsic factors are reported to be: regurgitation, recurrent stress-induced vomiting, certain psychosomatic disorders such as anorexia, bulimia and rumination, alcohol-abuse and GERD [1, 10-12]. One of the consequences of GERD is the erosive lesions that predominantly affect the enamel of the palatal surface of teeth [1].

GERD and other intrinsic factors can instigate the dental erosion since they may reduce the saliva pH (pH=5.5) to the levels below the critical pH in which hydroxyapatite crystals in the dental enamel dissolves.With a pH of less than 2.0; gastric reflux is potentially capable of causing dental erosion [1, 8, 10].

Attrition occurs at specific locations but erosion affects all surfaces of the teeth and can lead to discoloration, tooth sensitivity, shortened or rounded teeth, fractured teeth and even tooth loss [13].

Slight dissimilarities in the diagnosis of dental erosion around the word denote that the prevalence of the problem may not be exclusively similar in different countries [14].

Dentists can help in early diagnosis of patients who have unexplained dental erosions. With known potential impacts of GERD on dental tissues, dentists should often concern the refluxes and the subsequent GERD in taking the patient’s medical history. Collaboration of medical specialists in gastroenterology and dentists in the diagnosis and management of patients with GERD would inevitably improve the patients’ medical and dental health [13]. Regarding this burden, this study was planned to investigate the effects of gastroesophageal reflux disease (GERD) on dental erosion in a sample of Iranian population living in the Fars province.

## Materials and Method

A total of 140 patients ,with the average age of 30 to 50 years old, were recruited in this study. All participants were referred from the Motahari clinic to the department of endoscopy, Shiraz Namazi hospital. The study was approved by the ethics committee of the Shiraz University of Medical Sciences.

All participants were already eligible for the endoscopic examination. The requisite for the endoscopic examination was confirmed by their gastroenterologist. Two separate questionnaires were designed and submitted to the participants after giving adequate information about the study. The first questionnaire included the personal information and the clinical data regarding the gastric reflux. The second questionnaire included again, the personal information oral hygiene and dental evaluation. After completing the questionnaires and before endoscopy procedure, dental examination of the participants was performed by two blinded dentists. The dental examiners were completely trained regarding the differential diagnosis of dental erosion, attrition and similar lesions. The dental evaluation was considering the clinical caries, extracted teeth, restored teeth, replaced teeth and the gingival bleeding that was performed by a probe recommended by the WHO. Dental radiographs were not taken since the interproximal caries were not imperative regarding the aim of the current study. 

The endoscopy was then performed by the gastroenterologist .After definitive diagnosis of the disease by the gastroenterologist and based on the obtained data; patients were divided into three groups as follows:

1. Healthy (71 subjects)
2. Suspicious to reflux (38 subjects) and
3. Positive reflux (31 subjects).

The Chi- Square test was used for group comparison by employing SPSS (Ver. 15). Moreover, the cross tabulation test was performed to compare the qualitative variants and discover the correlations. The statistical significance was adopted as: p < 0.05.

## Results

**Figure 1:**
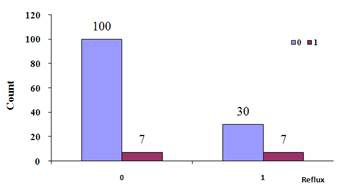
Chi-Square Bar Chart

The results of the Pearson Chi-square test, erosion- reflux cross tabulation, continuity correlation, likelihood ratio, Fisher’s exact test, linear- by- linear association are illustrated in table 1 and 2 and figure1 .

**Table 1:** Reflux* erosion Cross tabulation

		**Erosion**		**Total**
		**0**	**1**	
Reflux	Group 1 Count % within reflux % within erosion % of Total Residual	66 93.0% 52.4% 47.1% 2.1	5 7.0% 35.7% 3.6% -2.1	71 100.0% 50.7% 50.7%
	Group 2 Count % within reflux % within erosion % of Total Residual	36 94.7% 28.6% 25.7% 1.8	2 5.3% 14.3% 1.4% -1.8	38 100.0% 27.1% 27.1%
	Group 3 Count % within reflux % within erosion % of Total Residual	24 77.4% 19.0% 17.1% -3.9	7 22.6% 50.0% 5.0% 3.9	31 100.0% 22.1% 22.1%
Total	Count % within reflux % within erosion % of Total	126 90.0% 100.0% 90.0%	14 10.0% 100.0% 10.0%	140 100.0% 100.0% 100.0%

Group 1: Healthy

Group 2: Suspicious of having reflux

Group 3: Having reflux

**Table 2:** Chi-Square Tests

** **	**Value**	**df**	**Asymp. Sig. (2-Sided)**	**Exact Sig. (2-Sided)**	**Exact Sig. (2-Sided)**
Pearson Chi-Square	7.002a	1	.008	.015	.015
Continuity Correlation b	5.322	1	.021		
Likelihood Ratio	5.929	1	.015		
Fisher’s Exact Test					
Linear- by- Linear Association	6.952	1	.088		
N of Valid Cases b	140				

In the reflux positive group (Group 3, no= 31), a total of 7 individuals (22.6%) had erosions and 24 individuals (74.4%) did not show any erosions.This difference was statistically significant (p= 0.00).

In the reflux-suspicious group (Group2, no=38), 36 individuals (94.7%) had no erosions and only 2 participants (5.3%) showed dental erosions. This difference was statistically significant (p= 0.001).

The Cross tabulation test also showed that out of 71 healthy individuals (group 1), 5 individuals (7%) had dental erosions and 66 individuals (93%) did not show any dental erosion. 

In the first group, 5 patients (7%) had dental erosion whereas the number of subjects having dental erosion in the second and third group was 2 (5.3%) and 7 (22.6%) respectively (table 1). The results show that the higher percentage of erosions are seen in the reflux-positive group.

## Discussion

According to the Chi- square test results, the P-value was less than 0.05 in the group 3, indicating the statistical relationship between the reflux and the dental erosion. This result, in turn, means that the risk of dental erosions is increased in the patients with GERD (table 2, figure 1).

Gastric acid has destructive effects on the tooth structure and can be originated from both intrinsic and extrinsic sources. If the acid contacts the tooth longer than the time which could be tolerated by the dental enamel; the enamel will be demineralized and this process is named: dental erosion [15].

The loss of tooth material by this erosion is a dynamic procedure which contains both demineralization and remineralization. The etiology of dental erosion is a multipart assortment of the extrinsic and intrinsic elements [1, 16].

Various laboratory studies and many case-control and observational clinical trials have declared a positive- even if conflicting- relationship between GERD and dental erosion, both in adults and children [2].

In fact, the association of the dental erosion with GERD was first reported in 1933. However, apart from occasional published case reports, few studies had evaluated this association until the 1990s [2]. According to Böhmer et al. [17] 65.5% of individuals showed dental erosion with considering the abnormal pH less than 4.

In 2006, in a global evidence-based consensus named Montreal consensus, 44  doctors from 18 different countries voted to confirm the statement that the occurrence of dental erosions is increased in patients suffering from the GERD [2]. This was a high-grade consensus agreement (96%), however; only 42% of the voters were strongly agreed with the statement, 35% and 19% agreed with minor and major reservations respectively. The global consensus report also stated GERD as one of several potential aggravating cofactors [2].

In 2008, a systematic review enrolled by Pace et al. [18] analyzed 17 studies (observational and case- control) and showed a strong association between GERD and dental erosion. The median incidence of dental erosion in patients with GERD was reported 24%, and the median prevalence of GERD in adults with dental erosion was 32.5% and in children with dental erosions reported to be 17% [18].

Another systemic review, performed in 2009 by Tolia et al. found a higher prevalence of dental erosion in children with GERD compared with the healthy control group [19].

Regarding the dental erosion in children, dentists recognized the presence of this erosion in the children suffering from the gastric reflux and recurrent vomits many years ago [9]. In a study by Nazankocatus Ersin et al. [20], they discovered that GERD children were at higher risk of manifesting the dental erosion and caries compared with the healthy individuals. Their results were in agreement with the results of the current study on adult subjects. They also investigated the GERD effect on the saliva of 38 examined children and found that the salivary yeast and salivary streptococcus mutans colonization was apparently higher than the normal population.

In 2011, however, Wild et al. [21] presented a cross-sectional study of 59 children with ages between 9-17 years old with symptoms of GER and 20 asymptomatic children as the control group and did not find any association between the dental erosion and the GERD nor with the bacterial load. They concluded that future prospective researches are needed to find out the pathogenesis of dental erosion which is caused by GERD.

Another research in 2009 by Holbrook et al. [22] studied 249 Icelandic children and adults and found a significant association between the diagnosed GERD and dental erosion.The results are in line with the results of the current study.

A systematic review enrolled in 2011 by Firouzei et al. [8] , named SEPAHAN systematic review, found that 3 studies out of 10 original studies reported significant association between dental erosion and GERD in adults.

Firouzei et al. [8] concluded that GERD and dental erosion are strongly associated in adult population whilst they stated that this association is not very strong in young individuals. They confirmed the importance of early diagnosis and treatment of GERD in preventing the dental damage and tooth loss. The primary health care doctors, dentists and the gastroenterologists should be more concerned in this issue. The result yielded by the current study is in agreement with the conclusions of this research.

Different methods have been used in the studies related to the GERD such as esophageal pH monitoring (considered as the gold standard) and endoscopy and sometimes both techniques are employed to evaluate the reflux symptoms [8]. We could only use the endoscopy procedure in our study.

The location of dental erosion and its specificity could indicate the GERD and might help with the early diagnosis of this condition. It is stated that saliva has a protective role for dental tissues, contains chemoprotective elements and exhibits antimicrobial characteristics [1, 8].

Moreover, the qualitative and quantitative abnormalities of saliva have been linked to the pathogenesis of GERD [8]. Low buffering capacity with the normal flow rate in the GERD patients was reported and no study found to support that dental erosion might be linked with a decreased salivary flow [8].

By contrast, systematic reviews and reviews of literature describe many studies which show no significant associations between GERD and dental erosion but significant associations between GERD and xerostomia and halitosis [1-2, 8].

This is probably due to the fact that, in spite of the common belief of GERD as the major etiologic factor in the induction of dental erosion, it is difficult to compare the results of different studies due to the different scoring systems, samples, and examiners [1, 5, 8].

This study was performed to evaluate the association of oral manifestations, particularly (dental erosion)with GERD in Motahari clinic where many patients are referred from local and neighboring cities. 

Further randomized clinical trials and strong  meta-analysis studies are required to confirm the evidence-based relationship between GERD and dental erosion. 

In this study, 22.6% of individuals with positive GERD in endoscopy manifested the dental erosion and a significant statistical relationship was found between the GERD and the dental erosion. A collaboration of the medical and dental health care system is strongly recommended in the management of the patients with diagnosed GERD.

## Conclusion

Regarding the existing difference in the oral hygiene and diet of Iranian population, this study declared that the GERD patients are at higher risk of developing dental erosion compared to the healthy individuals in a sample of Iranian patients referred to the Motahari clinic, Shiraz, Iran.
